# Are We Driving Our Kids to Unhealthy Habits? Results of the Active Healthy Kids Canada 2013 Report Card on Physical Activity for Children and Youth

**DOI:** 10.3390/ijerph110606009

**Published:** 2014-06-05

**Authors:** Casey E. Gray, Richard Larouche, Joel D. Barnes, Rachel C. Colley, Jennifer Cowie Bonne, Mike Arthur, Christine Cameron, Jean-Philippe Chaput, Guy Faulkner, Ian Janssen, Angela M. Kolen, Stephen R. Manske, Art Salmon, John C. Spence, Brian W. Timmons, Mark S. Tremblay

**Affiliations:** 1 Healthy Active Living and Obesity Research Group, Children’s Hospital of Eastern Ontario Research Institute, 401 Smyth Road, Ottawa, ON K1H 8L1, Canada; E-Mails: rlarouche@cheo.on.ca (R.L.); jbarnes@cheo.on.ca (J.D.B.); rcolley@cheo.on.ca (R.C.C.); jpchaput@cheo.on.ca (J.-P.C.); mtremblay@cheo.on.ca (M.S.T.); 2 Active Healthy Kids Canada, 77 Bloor Street West, Suite 1205, Toronto, ON M5S 1M2, Canada; E-Mail: jencb@activehealthykids.ca; 3 Department of Health and Wellness, P.O. Box 488, Halifax, NS B3J 2R8, Canada; E-Mail: michael.arthur@bellaliant.net; 4 Canadian Fitness and Lifestyle Research Institute, 201-185 Somerset Street West, Ottawa, ON K2P 0J2, Canada; E-Mail: ccameron@cflri.ca; 5 Faculty of Kinesiology and Physical Education, University of Toronto, 55 Harbord Street, Toronto, ON M5S 2W6, Canada; E-Mail: guy.faulkner@utoronto.ca; 6 School of Kinesiology and Health Studies, and Department of Public Health Sciences, Queen’s University, Kingston, ON K7L 3N6, Canada; E-Mail: ian.janssen@queensu.ca; 7 Department of Human Kinetics, St. Francis Xavier University, P.O. Box 5000 (Courier 1 West Street), Antigonish, NS B2G 2W5, Canada; E-Mail: akolen@stfx.ca; 8 Propel Centre for Population Health Impact, University of Waterloo, 200 University Avenue West, Waterloo, ON N2L 3G1, Canada; E-Mail: steve.manske@uwaterloo.ca; 9 Ontario Ministry of Tourism, Culture and Sport, Hearst Block, 9th Floor, 900 Bay Street, Toronto, ON M7A 2E1, Canada; E-Mail: drfish@sympatico.ca; 10 Faculty of Physical Education and Recreation, University of Alberta, W1-16h Van Vliet, Edmonton, AL T6G 2H9, Canada; E-Mail: jc.spence@ualberta.ca; 11 Child Health & Exercise Medicine Program, McMaster University, 1280 Main Street West, Hamilton, ON L8S 4K1, Canada; E-Mail: timmonbw@mcmaster.ca

**Keywords:** motor activity/physiology, schools, transportation/methods, child, adolescent, humans

## Abstract

This article examines the time trends in patterns of school travel mode among Canadian children and youth to inform the Active Transportation (AT) indicator of the 2013 Active Healthy Kids Canada Report Card on Physical Activity for Children and Youth. The AT grade was assigned based on a comprehensive synthesis of the 2000 and 2010 Physical Activity Monitor studies from the Canadian Fitness and Lifestyle Research Institute and the 1992, 1998, 2005, and 2010 General Social Survey from Statistics Canada. The results showed that in 2013, AT was graded a D, because less than half of Canadian children and youth used only active modes of transportation to get to and from school. The proportion of Canadian children and youth who used only inactive modes of transportation for school travel increased significantly from 51% to 62% over the last decade. Children and youth from larger communities and those with lower household income levels were significantly more likely to use AT than those living in smaller communities and those in higher income households, respectively. In conclusion, motorized transport for school travel has increased steadily over the last decade across Canada. Regional and socio-demographic disparities should be considered in efforts to increase the number of children using AT.

## 1. Introduction

Physical inactivity is a global problem. According to an international survey, less than 20% of youth reported engaging in at least 60 min of daily physical activity of a moderate to vigorous intensity [1]. In Canada, only 7% of 5- to 17-year-olds are meeting the Canadian Physical Activity Guidelines [2]. Sources of physical activity that could once be counted on to ensure children and youth remain sufficiently active (e.g., walking or biking to/from school or other destinations and active outdoor play) are being engineered out of daily life and replaced by motorized travel and sedentary leisure activities (*i.e*., television, computer, and video game use). The net effect of these changes is that Canadian children and youth spend 62% of their waking hours engaged in sedentary behaviours [2].

Active transportation (AT; using non-motorized travel modes such as walking and cycling to/from school and other destinations) may offer an accessible and affordable solution to help young people increase their physical activity levels [3]. To date, most AT research has focused on travel to/from school. Systematic reviews have shown that children and youth who engage in AT for school travel spend significantly more daily time being physically active than their peers who go by motorized transport, even outside of the time spent traveling to and from school [4,5]. Despite the benefits associated with AT, evidence from other countries including Australia [6], Switzerland [7], and the United States [8] shows marked declines in the mode share (*i.e*., the proportion of individuals engaging in AT) for trips to/from school over the last few decades. In Canada, similar trends have been observed in the Greater Toronto Area (GTA) [9] and in Montréal [10]. For example, data from the Transportation Tomorrow Survey indicated significant decreases in the mode share of AT to/from school among youth aged 11–13 years (from 53.0% to 42.5%) and 14–15 years (from 38.6% to 30.7%) between 1986 and 2006 [11]. Interestingly, 58% of parents in the GTA reported walking to school when they were young, while only 28% reported that their children do so today [11]. Although these regional studies provide some indication of the AT trends in Canada, little is known about the temporal trends in AT at the national level.

Since 2005, Active Healthy Kids Canada (AHKC) has released the annual *Active Healthy Kids Canada Report Card on Physical Activity for Children and Youth* [12]. The Report Card consolidates current research knowledge to provide the most up to date and comprehensive assessment of the physical activity of children and youth in Canada. The Report Card serves as the basis for media attention, public debate, policy development, research proposals, academic publications, communications campaigns, funding decisions and general discourse [12]. The 2013 Report Card highlighted the connection between AT and overall physical activity levels of children and youth. Although the 2013 Report Card synthesized and graded research on 17 indicators related to physical activity in Canadian children, this article will focus solely on the AT indicator [3]. Limited data are available on time trends in AT among Canadian children and youth. Therefore, the specific purpose of this article is to report the time trends in patterns of AT among Canadian children and youth that were examined as part of the Report Card development process.

## 2. Methods

### 2.1. Report Card Methods

Active Healthy Kids Canada (www.activehealthykids.ca) employs a strategic partnership model to produce the annual Report Card. As such, partnerships have been established with the Healthy Active Living and Obesity Research Group (HALO) and ParticipACTION. HALO, at the Children’s Hospital of Eastern Ontario Research Institute (www.haloresearch.ca), conducted the comprehensive review of academic and non-academic literature, led the content development and review process, and wrote the long form version of the 2013 Report Card. ParticipACTION (www.participaction.com) contributed leadership on the communications and public relations strategy. As it has for the past nine years, Active Healthy Kids Canada funded, oversaw, managed, and disseminated the 2013 Report Card. The partnership was supported by an interdisciplinary Research Work Group, which consisted of 10 research and content experts from across Canada (invited by the Chief Executive Officer of Active Healthy Kids Canada on the recommendation of the Chief Scientific and Scientific Officers; the criterion for recommendation included expertise in one or more knowledge areas of the Report Card and geographic representation), was responsible for contributing data, reviewing content, and informing the grade assignment process.

Following the data gathering and synthesis process, the Research Work Group convened to evaluate the aggregated evidence and assign grades for each indicator. Key considerations included the quality of the compiled evidence and the presence of disparities (e.g., time-trends, geographic differences, and socioeconomic differences). Indicators were discussed until a consensus was reached using a letter-based grading scheme based on the proportion of children and youth meeting a defined benchmark or optimal scenario: A, 81%–100%; B, 61%–80%; C, 41%–60%; D, 21%–40%; F, 0%–20%; INC, incomplete data. The benchmark for AT was defined as the proportion of children and youth that engage in AT. The grading scheme also incorporated a plus-minus system depending on trends over time and the presence of, or reduction of disparities (*i.e*., race/ethnicity, disabilities, immigration status, socioeconomic status, geography, urban/rural setting, gender, and age). Further details on the full Report Card development process are available elsewhere [12].

### 2.2. Active Transportation Data

The 2013 grade for AT based on data collected in 2000 and 2010 as part of the Physical Activity Monitor (PAM) from the Canadian Fitness and Lifestyle Research Institute (CFLRI) [13,14], and to a lesser extent, in 1992, 1998, 2005, and 2010 by the General Social Survey (GSS) from Statistics Canada [15]. The PAM is an annual random-digit dialing telephone-based survey of a sample of Canadian adults collected over a 12-month period. The PAM tracks population level changes in physical activity patterns, and factors influencing participation (including socio-demographic information) in Canada. Each wave of the survey has a specific physical activity related focus; in 2000 and 2010 the PAM assessed parental perceptions of factors that support children’s physical activity, including use of AT. Each PAM was approved by the Human Participants in Research Committee of the York University Ethics Review Board. In addition, the 2010 PAM was approved by the Ethics Review Board of Health Canada. In the 2000 PAM, 2835 parents responded to a question about their child’s AT to/from school (52% response rate) [13]. Specifically, interviewers asked parents, “How does (Child) usually get to and from school? Does he/she walk, ride a bike, go by car, catch a bus or train, or go there some other way?” and prompted them to select the best response from these options. In 2010, 3699 parents responded to the same survey (38% response rate) [14]. The PAM sampling procedures are generally designed to target a representative sample of the adult population, as was the case in 2000. However, during the 2010 wave (N = 1797) the PAM sampling procedure was designed to represent the population of Canadian 5- to 17-year-olds according to province/territory of residence. Frequencies and prevalence estimates by sex and age were weighted to reflect sample design, however, in 2010 these were calculated using the Complex Samples cross tabulation procedure with 95% CIs [13,14]. To determine significant differences between estimates, 95% confidence intervals were calculated. In 2000, 62% of the total sample was aged 5–12 years and 38% was aged 13–17 years. The proportion was comparable in 2011, with 59% of the total sample aged 5–12 years and 41% aged 13–17 years.

The GSS is an annual random-digit dialing telephone-based cross-sectional survey of a probability sample of Canadians aged 15 and over from all provinces [15]. It was started in 1985 to examine social trends, living conditions and the well-being of Canadians. The sample size of the survey increased in 1999 from 10,000 to 25,000. The Time Use survey is a quinquennial wave of the GSS that monitors trends on the living conditions and well-being of Canadian families. Data on AT to/from school for adolescents aged 15–17 years were collected from January to December in 1992, 1998, 2005, and 2010 in the “Commute to Work” module of the Time Use GSS. Of relevance to the Report Card, interviewers asked adolescent participants, “Last week, how did you get to school?” and prompted them to select all responses that apply from a menu of active and inactive modes (car, truck or van-as driver; car, truck or van-as passenger; public transit (e.g., bus, streetcar, subway, light-rail transit, commuter train, ferry); walked to school; bicycle; motorcycle; taxicab; attends school at home; other; don’t know; or refusal). A custom tabulation of relevant data for the 15 to 17 year old age group from the GSS was obtained from Statistics Canada. A more comprehensive description of the PAM and GSS-Time Use methodologies are available elsewhere [13,14,15].

## 3. Results

**Figure 1:**
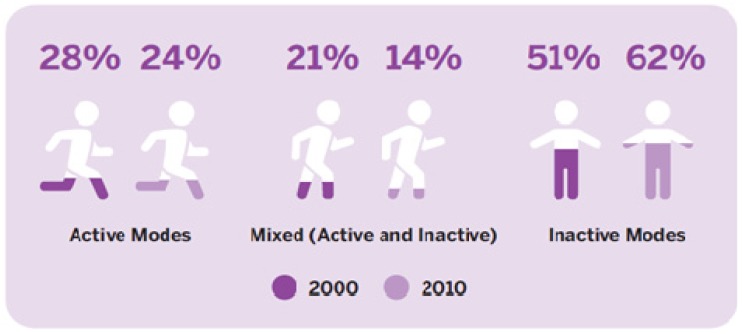
Usual modes of transportation among Canadian children and youth to/from school between 2000 and 2010 [3].

Source: Canadian Fitness and Lifestyle Research Institute Physical Activity Monitor, 2000, 2010.

In the 2013 AHKC Report Card, AT was assigned a ‘D’. The AT grade was informed to a large extent by the PAM, according to which, 24% (CI, 22.2%–25.9%) of children and youth used only AT for trips both to and from school in 2010 [16]. The 2010 PAM data did not differ significantly from 2000 when 28% (CI, 25.2%–31.0%) of parents reported AT as the only mode of school travel used by their children and youth [16]. Over the same period, parent reports indicated a statistically significant increase in the proportion of children and youth that used only inactive modes of transportation for school travel [16]. In 2000, 51% (CI, 47.5%–53.8%) of young people were driven to school (30% by bus or train, 15% by car, 6% by a combination of inactive modes); by 2010 the proportion of inactive travelers had increased to 62% (CI, 60.3%–64.4%) (34% by bus or train, 24% by car, 4% by a combination of inactive modes) [16] (Figure 1). The proportion of children and youth who traveled to/from school using a combination of active and inactive modes decreased significantly over time (21%, CI, 19.0%–23.9%, in 2000, 14%, CI, 12.2%–15.1% in 2010) [16].

Detailed examination of PAM transportation mode data was conducted for age, geographic and socio-economic-related variables. Younger children (aged 5–12 years) were more likely to use AT and less likely to travel by inactive modes than older children (aged 13–17 years) in the 2000 PAM, however these age related differences were not significant in the 2010 PAM (25% of 5–12 year olds and 23% of 13–17 year olds used only active modes; 62% of 5–12 year olds and 63% of 13–17 year-olds used only inactive modes for school travel [16]). Within inactive travel, younger children were significantly more likely to travel exclusively by car in 2011 (28%) than in 2000 (15%) [13,16].

**Figure 2:**
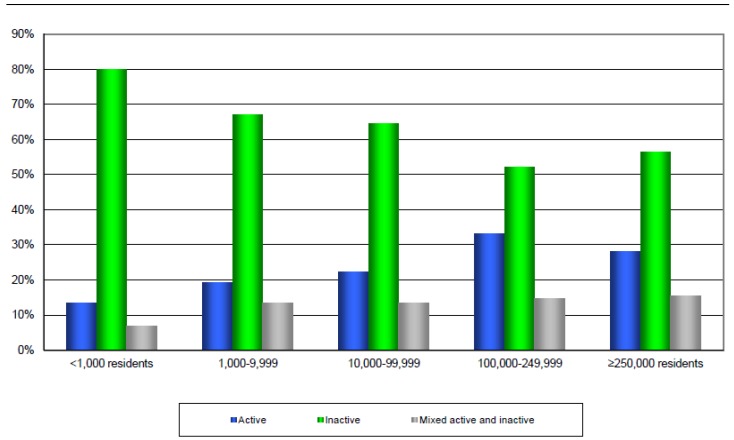
School travel mode used by Canadian children and youth stratified by community size. [16].

Source: Canadian Fitness and Lifestyle Research Institute Physical Activity Monitor, 2010.

In terms of geography, the proportion of active school travelers varied with community population size in 2010 [16] (Figure 2). Specifically, in communities with fewer than 1000 residents, 13% of children used only AT, while 80% travelled by only inactive modes [16]. In contrast, in communities of 100,000 to 249,999 residents, 33% used AT for school travel, while 52% used only inactive modes [16]. Regarding socio-economic status, children and youth from the lowest household income bracket (<$50,000/year) were significantly more likely to use active modes of transportation (28%) than their more affluent ($80,000–$100,000/year) counterparts (18%, respectively). They were also less likely to travel only by inactive modes (58%) than their more affluent counterparts (72%) [16]. This pattern of AT mode share is consistent with data gathered in the 2000 PAM [13]. A significantly greater proportion of parents from communities with fewer than 1000 residents and families in the highest income bracket (>$100,000) reported that their children only use sedentary modes of transportation for school travel in 2000, while children and youth in larger communities and those with lower family income were more likely to only use AT [13,16]. It is worth noting that there were no differences in AT use by gender or by age [16].

**Figure 3:**
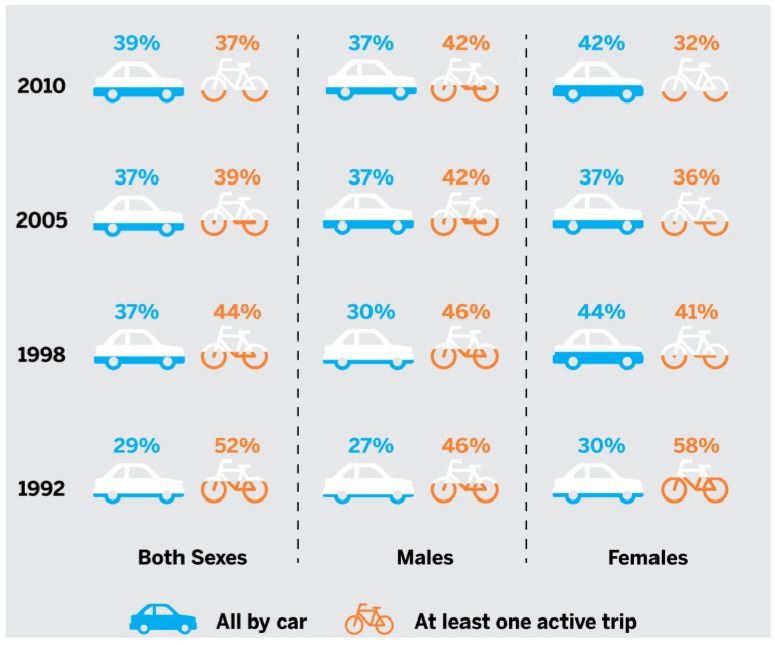
The percentage of 15- to 17-year-olds in Canada who take all their daily trips by car and who take at least one daily trip using active transportation between 1992 and 2010 [3].

Source: Statistics Canada General Social Survey, 1992, 1998, 2005, 2010.

In 2013, the AT Report Card grade was also informed by 20-year trends uncovered by the Time Use GSS. According to the GSS Commute to Work module data, the proportion of Canadian youth who used AT for at least one school trip per day has declined by 13 percentage points between 1992 (52%) and 2010 (39%) [17] (Figure 3). In comparison, the proportion of youth who take all of their school trips by car has increased steadily during the same period, with a 10 percentage point increase observed between 1992 (29%) and 2010 (39%) [17] (Figure 3). Finally, 45.5% of youth reported that they travelled by public transit for at least 1 daily trip during the survey week in 2010 [17].

## 4. Discussion

Globally, AT for school travel is on the decline among children and youth (e.g., [6,7,8]). However, a lack of national data collected at regular intervals on AT has prevented commentary on temporal trends in Canada. Recently released nationally representative data on the AT levels of Canadian children and youth collected in 2000 and in 2010 by the CFLRI presented in this paper indicates a significant increase in the proportion of children and youth who relied solely on motorized school travel over the last decade [16]. This pattern is supported and extended by observations reported in the GSS, which suggests that a trend of increasing motorized travel and decreasing AT for school trips has been in progress for nearly 20 years [17]. Nationwide reports of increased motorized school travel in Canada are in line with regional observations in the GTA [9] and in Montreal [10].

It is possible that the disappearance of age related differences in AT and inactive travel observed from 2000 to 2010 by the PAM is accounted for by the shift toward younger children being driven to school by car in 2010. However, without contextual data it is difficult to speculate as to why more children are being driven. Statistics Canada data show that most Canadians are urban dwellers and that this has remained stable across the period of time covered by the current study (77% in 1991; 81% in 2011) [18]. In this case, the term ‘urban’ includes sub-urban dwellers, making it impossible to determine if the distance between home and school has expanded due to a possible sub-urban migration. Another possibility is that more parents of younger children may be choosing to send their children to specialized schools (e.g., private, Catholic, or English schools as in Quebec). Indeed, Quebec data show that specialized schools tend to be further away from children’s homes and the children attending those schools are less likely to engage in AT [10].

Socio-demographic differences in AT mode share observed in the 2000 PAM, which continued into 2010 indicate that broad efforts to increase AT are unlikely to be successful in reversing this trend. Instead, given the diversity of influential factors at play, interventions must be tailored to the local context if they are to make a difference [19,20]. For example, enduring disparities in children’s and youth’s AT use according to degree of urbanization were observed by parents in 2000 and 2010 [14,16]. Residents of larger communities by population were more likely to report that their children and youth usually engaged in AT than those in communities with fewer inhabitants [14,16]. This difference may be partly attributable to the greater distance between home and school for many children living in rural areas [21]. A similar pattern has been observed in regional Canadian research showing higher rates of AT to/from school in urban compared to suburban areas [9,19,20]. Urban areas generally have a greater population density, which may translate into shorter home-school distances for the average child [22]. This could potentially explain the greater use of AT in larger communities. In the GTA, youth living in urban and inner-suburban areas were more likely to report use of AT than their outer sub-urban counterparts [19,20,23]. Furthermore, it is worth mentioning that Rainham and colleagues found that AT is the single most important source of daily moderate-to-vigorous physical activity for youth living in urban areas [23].

The PAM data also indicated disparities in rates of AT to/from school according to socioeconomic status (SES), with children from lower income families being more likely to engage in AT [16]. This finding is consistent with previous studies [9,19,24,25,26,27], including observations from the National Longitudinal Survey of Children and Youth [26]. This disparity is possibly explained by data which show that low SES children and youth tend to live closer to school and their parents are less likely to have access to a vehicle [8]. In Swiss research, children were significantly more likely to be driven to school in 2-car households [7]. Interestingly, when considering physical activity more broadly, an increased likelihood of physical inactivity among youth was associated with low material wealth and perception of low family wealth [28], making AT a particularly important source of physical activity for low income children and youth. Unfortunately, there is also evidence of disproportionate exposure to unsafe traffic environments in urban and low-income neighbourhoods [27], possibly increasing the risk of injury.

Given that participation in active school travel varies regionally and also across different types of space or neighbourhood [19], there is no ‘one size fits all’ solution to promoting greater AT. Interventions must consider local contextual factors and School Travel Planning (http://www.saferoutestoschool.ca) is one model for developing school specific plans to increase AT [29]. School Travel Plans are based on travel demand management principles and are largely a non-coercive form of travel demand management at the trip-end (school location) where school specific interventions are proposed and developed to change mode choice (e.g., shift from driving to walking) and improve safety. Depending on need and context as informed by members of the school community (e.g., students, parents, teachers), interventions may include enforcement of speed limits, the development of walking school buses, or education initiatives [29]. Schools may also consider partnering with local businesses willing to allow parking for busses and private vehicles. Walking school busses could then be arranged from those locations to provide children an opportunity to engage in AT for part of their school trip [30]. Preliminary results indicate reductions in auto-use for school travel following school travel plan implementation in Canada [29]. However, school travel planning traditionally targets the school environment. Features of the built environment, from school siting within neighbourhoods to features around the home also play a significant role in the school travel mode decision-making process [5,31]. Accordingly, broader policy based approaches are required in creating the social and physical conditions conducive to AT more equitably across neighbourhoods.

There are limitations inherent to the Report Card development process as well as the PAM and GSS surveys that warrant discussion. The grade assignment process is only as robust as the data on which it is based and in 2013 the AT grade was based on the PAM and GSS data. These surveys rely entirely on parental proxy-reports (PAM) of a usual week of school travel and adolescent self-reports (GSS) of school travel last week, which introduces possible biases and inaccuracies. A combination of accelerometers and global positioning systems could address these biases while allowing a more precise quantification of the relationship between AT and physical activity. Nevertheless, these surveys provide us with nationally-representative data, which are, to our knowledge, the first examination of time trends in AT to/from school at the national level in Canada. Although these data are not directly comparable for several reasons (e.g., different age groups were sampled; self-report was used by the GSS and parental proxy report was used by the other; the PAM asked about the child’s usual mode of school travel, while the GSS asked about all modes of school travel engaged in last week), they do provide a cohesive indication of school travel patterns of children and youth.

A further limitation relates to the lack of data on the distance between home and school. Regional data show that distance between home and school is the major predictor of AT for school travel. Unfortunately, it is not possible to draw conclusions about the distance between home and school, or to report on any possible changes that may have occurred over time as distance between home and school were not collected in the GSS, and were only collected for active travelers at one time point in the 2011 PAM.

A key research gap highlighted in the 2013 Report Card is the lack of Canadian data on AT to destinations other than school. Given that the strongest barrier to AT to/from school is the distance between home and school [31,32], other nearby destinations such as parks, shops and sport fields may provide important, yet overlooked opportunities to engage in AT [29]. There is a clear need for research investigating the correlates of AT to a broader range of destinations to inform the development of novel strategies to promote AT.

## 5. Conclusions

In conclusion, this paper highlights the changing patterns of travel mode to/from school that have been observed among Canadian children over the past 10–20 years. Nationally representative surveys suggest the proportion of Canadian children and youth that rely solely on motorized school travel is on the rise. The increase is more pronounced for young people who live in smaller communities and those from higher SES households. Context-specific strategies are needed to facilitate opportunities for AT for children and youth to increase their use of AT.
